# Prediction of Clinical Trajectory in HCV-Related ACLD after SVR: Role of Liver Stiffness in a 5-Years Prospective Study

**DOI:** 10.3390/v16091439

**Published:** 2024-09-10

**Authors:** Filomena Morisco, Alessandro Federico, Massimo Marignani, Flavia L. Lombardo, Valentina Cossiga, Luisa Ranieri, Mario Romeo, Marina Cipullo, Paola Begini, Alessandra Zannella, Tommaso Stroffolini

**Affiliations:** 1 Department of Clinical Medicine and Surgery, University of Naples “Federico II”, 80131 Naples, Italy; valentina.cossiga@gmail.com (V.C.); luisa.ranieri92@gmail.com (L.R.); 2 Departmental Program “Diseases of the Liver and Biliary System”, AOU Federico II, 80131 Naples, Italy; 3 UNESCO Chair: Environment, Resources, and Sustainable Development, University of Naples “Federico II”, 80123 Naples, Italy; 4 Hepato-Gastroenterology Unit, University of Campania Luigi Vanvitelli, 80138 Naples, Italy; alessandro.federico@unicampania.it (A.F.); mario.romeo@unicampania.it (M.R.); marina.cipullo@studenti.unicampania.it (M.C.); 5 Department of Digestive and Liver Disease, S. Andrea University Hospital, 00189 Rome, Italy; mmarignani@hotmail.com (M.M.); paolabegini@virgilio.it (P.B.); alezannella@gmail.com (A.Z.); 6 Department of Gastroenterology and Hepatology, Regina Apostolorum Hospital, 00041 Rome, Italy; 7 National Center for Disease Prevention and Health Promotion, Italian National Institute of Health, 00161 Rome, Italy; flavia.lombardo@iss.it; 8 Department of Tropical and Infectious Diseases, Policlinico Umberto I, 00161 Rome, Italy; tommaso.stroffolini@hotmail.it

**Keywords:** direct-acting antiviral, SVR, LSM, HCC, HCV

## Abstract

The prediction of liver-related events (LRE) after sustained virological response (SVR) in HCV-advanced chronic liver disease (ACLD) patients is crucial. We aimed to evaluate incidence and risk factors of LRE in HCV-cirrhotic patients after SVR and to assess dynamic changes of liver stiffness in participants without LRE at the end of follow-up. We enrolled 575 consecutive patients with HCV-ACLD treated with DAAs and followed up for 5 years after SVR12. Overall, 98 (17%) patients developed any type of event, and HCC was the most frequent LRE. The incidence rate was 1.6 per 100 person-years (p/y) for both HCC and hepatic decompensation. Baseline LSM ≥ 20 kPa was the only independent predictor of hepatic decompensation, while LSM ≥ 20 kPa and male sex were independent predictors of HCC development. Among the 341 participants without LRE and with paired LSM, any LSM reduction was observed in 314 (92.1%), and half of them showed a decrease of LSM ≥ 20%. Among patients without LRE, 27.3% of participants without ≥20% LSM decrease at 2 years achieved the 5-year goal; in contrast, 31.6% of participants with ≥20% LSM decrease at 2 years lost it at 5 years. These findings provide evidence that baseline LSM is a tool to stratify patients at risk of developing LRE; the dynamic changes of LSM value suggest the need for monitoring this parameter over time.

## 1. Introduction

The sustained virological response (SVR) represents a relevant endpoint in the treatment of patients with chronic hepatitis related to HCV infection, with a consequent improvement of fibrosis rate progression, morbidity, and mortality [1,2,3]. For patients with advanced chronic liver disease (ACLD), outcomes after SVR appear to be less favorable, with a persistent residual risk of developing liver-related events (LRE), strictly linked to the patient’s pre-treatment history [4]. Available literature shows that in patients with compensated ACLD achieving SVR, decompensation incidence rate is about 2.3/100 person years (p/y) [5], while the rate of hepatocellular carcinoma (HCC) occurrence is 2.45 p/y [6]. Therefore, the clinical challenge for physicians after SVR is to accurately identify this subset of at-risk patients. In a previous study from our group, we have described that baseline liver stiffness measurement (LSM) was an independent predictor of LRE development post-SVR [7]. In detail, subject with LSM ≥ 20 kPa showed a 4-fold higher risk of liver decompensation and a 7-fold higher risk of HCC as compared to the group with baseline LSM < 20 kPa.

In association to liver-stiffness values, other risk factors for the development of post-SVR LRE, such as diabetes mellitus and male sex, were also identified and a specific management has been proposed for patients with these characteristics [8]. Conversely, many issues regarding post-SVR fibrosis behavior remain open. Few studies, focusing on fibrosis regression, have followed patients for 24–36 months, that probably represents a too-short period to track long-term changes [8,9] and to evaluate the variability of fibrosis regression degree over time and the clinical and the biological factors associated to these variations [10]).

The present prospective study has evaluated the role of basal LSM as a tool to identify patients at risk of developing LRE after 5 years of SVR achievement and the dynamic LSM changes, if any, over time.

## 2. Materials and Methods

### 2.1. Study Design and Target Population

This is an extended prospective longitudinal study conducted for up to 5 years in a multicenter cohort of HCV patients followed-up in three hepatology tertiary centers in Italy (University of Naples Federico II, University of Naples Luigi Vanvitelli, Sant’Andrea University Hospital of Rome). Consecutive patients with HCV-related ACLD starting DAA therapy between September 2014 and June 2018 were enrolled. Exclusion criteria were history of HCC, prior liver transplantation, HBV and/or HIV co-infections, history of alcohol consumption (previous and/or continuative use), failure to achieve SVR12, and Child–Pugh C score, since in this latter group DAA treatment was not reimbursed by the Italian National Health System outside the liver transplantation waiting list [11]. ACLD was defined histologically (METAVIR F4) [12], non-invasively (LSM > 11.9 kPa) [13], or based on the presence of the peculiar clinical, biochemical, and ultrasound signs [14]. Type of antiviral treatment protocol was selected and administered at the physician’s discretion, according to drug label and international recommendations [15,16]. All participants signed informed consent before the enrollment. The study was conducted in accordance with the Declaration of Helsinki. The protocol was approved by the ethical board of the promoting center (Federico II University of Naples, n. 245/13).

### 2.2. Follow-Up and Measurements

Follow-up began at SVR12, which was defined as undetectable HCV-RNA at 12 weeks after the end of therapy using the Cobas AmpliPrep/Cobas TaqMan (Roche Molecular Diagnostics, Pleasanton, CA, USA; lower limit of detection 15 IU/mL).

Enrolled patients were followed up for up to 5 years after achieving SVR12 with regular biannual clinical, laboratory, and instrumental follow-up. HCC surveillance was performed with ultrasound every 6 months according to standard clinical practice. If liver focal lesions were identified by ultrasound, HCC was confirmed by imaging (computed tomography and/or magnetic resonance imaging) and/or biopsy examination according to international guidelines [17]. This program was hindered by the COVID-19 lockdown for most of the year 2020, even if a telemedicine approach was offered to all followed-up patients. Baseline demographic and clinical characteristics of patients, treatment outcomes, types, and timing of event occurrences were recorded in a shared web database. At the end of the observation period, cumulative and separate analysis were performed in patients who did and did not develop LRE.

### 2.3. Liver Stiffness Measurement

LSM was performed using transient elastography with Fibroscan (Echosense, Paris, France) by a single experienced operator in each center and according to the usual standard procedure, at 3 time points: at baseline (month 0), at 2 years (month 24), and at 5 years (month 60). The results were expressed in kilopascals (kPa) with a range from 2.5 to 75 kPa. Interquartile range (IQR) was defined as an index of the intrinsic variability of LSM. Only those measurements with more than 10 successful acquisitions, with a success rate of at least 60% and an IQR lower than 30%, were classified as valid and taken into consideration for statistical evaluation [18].

### 2.4. Study Outcomes

The main study outcome was the analysis of LRE developing after the achievement of SVR. Liver-related events were defined as:

- New onset of: (1) ascites; (2) variceal bleeding; (3) spontaneous bacterial peritonitis; (4) hepatic encephalopathy; (5) liver transplantation: (6) death for Child–Pugh A and B patients;
- Occurrence of de novo HCC;
- In Child–Pugh B patients, worsening of a pre-existing symptom of decompensated cirrhosis (i.e., need for an increased dose of diuretics, addition of rifaximin for hepatic encephalopathy, or hospital admission for a new liver failure event) were also considered as LRE.

Any LRE developing during follow-up was registered in the web database. Mortality was also registered, as either liver-related or not liver-related. The at-risk period for each subject was defined by the time from the SVR12 until the event onset. Events occurring before the end of antiviral treatment were not included in the analysis. Data were censored at the end of the study if no event had occurred and if individuals were lost during follow-up. Patients lost at follow-up were registered as alive until the last day of observation.

### 2.5. Statistical Analysis

Baseline and clinical characteristics are expressed as mean and standard deviation (SD) for continuous variables and compared by *t*-test or Kruskal–Wallis test if not normally distributed; qualitative and categorical variables are presented as frequency and evaluated by the chi-square test; a *p*-value < 0.05 was considered statistically significant.

Time to event for each outcome was calculated as the time from SVR12 achievement to the first onset of event. Incidence density rates of LRE per 100 person-years were provided with 95% confidence intervals (95% CI). The Kaplan–Meyer method was used to estimate the cumulative incidence of events. Comparison of survival curves between groups was performed using the log-rank (Mantel–Cox) test. Univariate and multivariate Cox regression models were used to identify baseline variables associated with the development of any LRE or HCC. Variables with a threshold p-value lower than 0.10 at univariate analysis were included in the multivariate model. The assumption of proportional hazards was tested using Schoenfeld residuals. The optimal baseline liver-stiffness cutoff was determined by Youden’s index method maximizing the difference between true positive rate and false positive rate. Based on Youden’s method, the optimal stiffness values for the prediction of hepatic decompensation and for the occurrence of HCC are 19.8 kPa and 19.3 kPa, respectively. Since these thresholds are very close to the value of 20 kPa (widely used in literature), this latter value (20 kPa) was maintained in subsequent analysis. The predictors of improvement in LSM, defined as a reduction ≥ 20%, were evaluated thought logistic regression. Statistical analyses were performed using STATA 17.0 statistical software (StataCorp, College Station, TX, USA).

## 3. Results

### 3.1. Patients’ Characteristics

The baseline characteristics of 575 participants achieving SVR12 are summarized in Table 1. The male/female ratio was 1.1, and the mean age was 64.1 ± 12 years. Genotype 1 was predominant and present in 79.8% of cases. Child–Pugh stage was A in 94.4% of our population, while 5.6% of patients were in stage B. Mean liver stiffness at baseline was 19.2 ± 7.3 kPa, and measurements were performed in 547/575 participants (95.1%).

### 3.2. Incidence of Liver-Related Events

The mean period of observation after SVR12 achievement was 4.6 years (Standard Deviation 1.0 year) for a total of 2676 person/years. During the study period 29 out of 575 (5.0%) patients were lost to follow-up (18 during the first 2 years, three during the third year, six and two during the fourth and fifth year, respectively). Table 2 reports the incidence rate per 100 person-years of the main events during follow-up. Overall, 98 (17.04%) patients developed events of any kinds, and mortality rate was of 8.8% (3.0% for liver-related cause and 5.8% for non–liver-related causes). HCC was the most frequent LRE (41 patients, 7.1%), followed by ascites (29 participants, 5%), and upper gastrointestinal bleeding (16 patients, 0.2%). Incidence rate was 1.6 per 100 p/y for both HCC occurrence and for liver decompensation. Among the 51 that died during follow up, 18 (35.3%) experienced an episode of liver decompensation, while 10 (19.6%) developed HCC. Moreover, 18 out of 41 participants with HCC (43.9%), also had a liver-decompensation event.

### 3.3. Basal Predictors of Liver-Related Events

**Figure 1:**
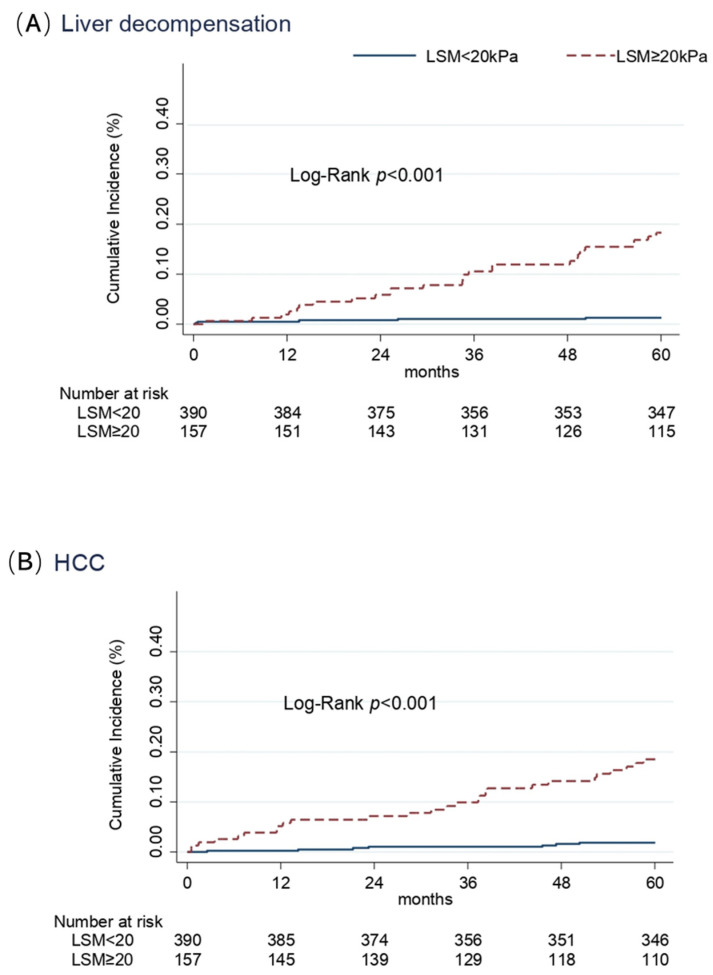
Cumulative incidence of liver decompensation (**A**) and HCC (**B**) in cirrhotic patients according to the value of stiffness at baseline (Kaplan–Meier estimates).

Variables independently associated with LRE (decompensation and HCC), in patients with ACLD who achieved SVR, are shown in Table 3. After adjusting for the confounding effect of each variable, selected on the basis of their association in the univariate Cox model, LSM ≥ 20 kPa (HR 13.5; 95% CI 5.2–35.3) was the only independent predictor of liver decompensation, while liver stiffness ≥ 20 kPa (HR 9.2; 95% CI 4.0–21.3) and male sex (HR 3.6; 95% CI 1.6–8.0) were both independent predictors of HCC development. Although the effects of age were found to have an HR of 2.2 and 2.0 for liver decompensation and HCC, respectively, these associations did not reach statistical significance at the 5% level. The cumulative incidences of liver decompensation and HCC according to the basal LSM are summarized in Figure 1. Incidence rate of liver decompensation was higher in the group with basal LSM ≥ 20 kPa as compared to patients with pre-treatment LSM < 20 kPa: 4.2 (95% CI 2.9–6.1) vs 0.3 (95% CI 0.1–0.7) per 100 p/y (Rate Ratio 15.5, *p* < 0.001) (Figure 1A). Similarly, HCC incidence rate was 10 times higher in patients with basal LSM ≥ 20 kPa than in participants with basal LSM < 20 kPa: 4.4 (95% CI 3.0–6.3) vs. 0.4 (95% CI 0.2–0.8) per 100 p/y (Rate Ratio 11.4, *p* < 0.001) (Figure 1B).

### 3.4. Dynamics of Liver Stiffness Measurements

**Figure 2:**
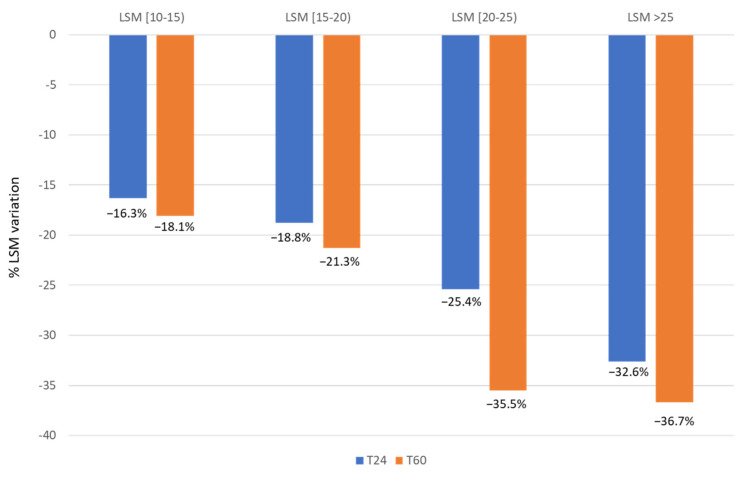
Dynamics of liver stiffness in patients without LRE at T24 and T60.

Overall, 477 patients did not experience LRE during the follow-up. Among them, 341 (71.5%) had LSM measure performed at T0 and T24, and 338 had available measure also at T60. In this subgroup, mean LSM at SVR12 was 16.9 ± 4.2 kPa, while it decreased to 13.0 ± 4.0 kPa at the end of follow-up (T60). Overall, LSM reduction of any magnitude was observed in 314 (92.1%) of patients, with a mean reduction of 4.4 kPa (range 1–39 kPa). A stable stiffness (intended as a stiffness increase of less than one point) was observed in 10 patients (2.93%), while a worsening of stiffness (intended as any stiffness increase) was observed in 17 patients (4.99%). Among the 314 patients with LSM reduction at the end of the follow-up, half (157 patients; 50%) showed a decrease of LSM ≥ 20%, while only 17 patients (5.4%) achieved a reduction of LSM to less than 6 kPa. LSM was performed in 338 patients at three time-points: SVR12 (T0), at 24 months after SVR12 (T24), and at the end of follow-up (T60). Stratified by baseline LSM, patients with LSM ≥ 25 kPa showed a greater reduction of LSM (−32.6% at T24 and −36.7% at T60), than those with LSM between 20–25 kPa (−25.4% at T24 and −35.5% at T60), those with LSM between 15–20 kPa (−18.8% at T24 and 21.3% at T60), and those with LSM between 10–15 kPa (−16.3% at T24 and −18.1% at T60) (Figure 2). We also analyzed the baseline characteristics of 338 subjects with paired LSM (at baseline and at end of follow-up) and without LRE during the 5 years’ follow-up, stratifying this subgroup according to the achievement of LSM reduction ≥ 20%. We did not find significant differences in age, sex, and viral genotype in the patients with LSM reduction ≥ 20% at 5 years compared to patients without LSM decrease > 20%). The only difference observed between groups was in LSM at baseline. These findings were confirmed by multivariate logistic regression, and the only factor associated with LSM reduction > 20% at 5 years of follow-up was the LSM at baseline (OR 1.2, 95% CI 1.1–1.3; *p* < 0.001).

**Figure 3:**
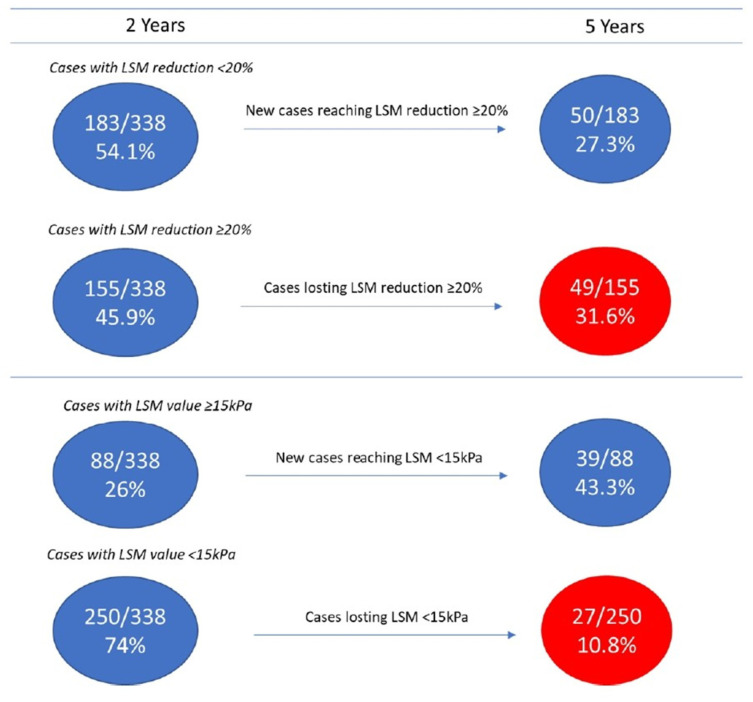
Changes over time in fibrosis degree from 2 to 5 years of follow-up in SVR patients without LRE.

Among patients without LRE, interesting changes were observed over time when comparing LSM values at 2 and 5 years of follow-up: 27.3% (50/183) of patients not reaching LSM decrease ≥ 20% at 2 years reached this target at 5 years; interestingly, 31.6% (49/155) of patients reaching the ≥20% LSM decrease at 2 years, lost it at 5 years (Figure 3). Changes over time were observed even using a LSM cut-off of 15 kPa: a further 44.3% (39/88) of patients, still above this cut-off at 2 years, were still below this cut-off at 5 years; conversely, 10.6% (27/254) of patients below this cut-off at 2 years, at 5 years showed a LSM value higher than this cut-off (Figure 3).

## 4. Discussion

This study of a 5-year follow-up after SVR evidences the following issues:

- SVR patients at the highest likelihood of lack of LRE are females and those with basal LSM < 20 kPa who have a 10-fold lower incidence of LRE at 5 years after SVR as compared to males and those with a basal LSM ≥ 20 kPa.
- The magnitude of fibrosis regression in SVR patients without LRE at 5 years depends on the basal LSM; results are higher in patients with more severe liver fibrosis at baseline. The reduction of LSM, at 5 years after SVR, is between 18.08% and 37.2%.

The only variable associated with the likelihood of at least 20% decrease of LSM at 5 years’ follow-up in SVR patients without LRE results is the basal LSM; while age, sex, and genotype do not have an impact on fibrosis regression.

HCC resulted in the most frequent LRE after SVR with an incidence rate of 1.6 per 100 p/y, in agreement with the 1.5 per 100 p/y rate reported by other studies [19,20]. Conversely, our finding is lower than the incidence rate of 2.45 per 100 p/y recently observed in a large Italian cohort [6], probably reflecting a more advanced stage of liver disease among this latter population (LSM > 20 kPa in 47.5%, Child–Pugh B in 14.9% and cases with previous liver decompensation in 10.9%).

Ascites is the second most frequent complication after HCC, with an incidence rate of 1.6 per 100 p/y, slightly higher than that observed in other similar cohorts (0.3–0.6/100 p/y) [19].

Overall, this study, with a follow-up extended to 5 years, confirms that basal LSM ≥ 20 kPa represents a strong predictor of HCC and liver decompensation occurrence, as already identified in our previous work, which had a limited 24 months’ follow-up after SVR [7]. This result is also in keeping with that reported in multivariate analyses conducted in large multicenter European cohorts [7,19,20,21,22] reflecting the good correlation with hepatic venous pressure gradient (HVPG). As also stated in the Baveno VII report, the cutoff of LSM ≥ 20 kPa is highly predictive of clinically significant portal hypertension, liver decompensation, and unfavourable outcomes [23,24].

Even if the present findings don’t represent a novelty, they confirm in a longitudinal study design data mostly provided by retrospective studies [25], which are usually affected by selection and survival biases. Moreover, this survey is to date one of the few reports with a long follow-up of 5 years. Currently, only one survey performed in a single center in the North of Italy had a similar duration of follow-up [4].

An interesting aspect of this study is related to the dynamics of LSM in patients who did not develop LRE at 5 years. A recent paper, conducted in a very large cohort of HCV patients successfully reaching SVR, strengthens the relevance of repeated LSM in the prediction of clinical outcomes [24]. This study enrolled 2508 patients with chronic liver disease with at least two LSM. The Authors found that patients with ACLD (compensated or decompensated) had a greater decrease in the median LSM as compared to non-ACLD patients. In detail, a LSM reduction ≥ 20% occurred in 26.2% of non-ACLD, 55.9% of cACLD, and 51.0% of dACLD (24. In our previous study, available data showed stiffness reduction, as expected, even if data were characterized by a high grade of heterogeneity). The magnitude of decline in LSM was incremental over time after completion of the therapy, but this event was not linear and not equal in all patients, and higher decreases were observed among patients with basal LSM > 25 kPa. A recent systematic review of the literature, analyzing 24 studies involving HCV patients treated with DAA, showed that the decline in LSM was higher in patients with cirrhosis as compared to those with chronic hepatitis [26]. Although our cohort was composed of patients with ACLD and the severity of liver fibrosis was highly heterogeneous, in keeping with the previously cited findings, we observed a more pronounced decline in LSM in patients with more severe liver stiffness at baseline. It has to be underlined that, with the extended follow-up to 5 years, we have eliminated the bias of LSM reduction due to a decrease of inflammation obtained after viral eradication [11].

Finally, the dynamic changes in LSM values at 2 and 5 years of follow-up deserve comments. Nearly one-third (27.3%) of patients who failed to reach the LSM decrease ≥ 20% at 2 years of follow-up obtained this target at 5 years. This is a positive and welcomed finding, suggesting a continuous regression of fibrosis even years after the SVR achievement. This finding confirms previous studies showing that LSM continued to decline in a considerable proportion of patients with advanced liver disease after HCV eradication [27,28,29].

Conversely, 31.6% of patients who had obtained this beneficial target at 2 years lost it at 5 years. This is a new and unexpected finding that is difficult to explain. A possible reason might be the potential role of unmeasured hepatotoxic factors (i.e., risky alcohol intake after HCV eradication, drugs, diabetes, high BMI, diet, lifestyle, and so on). The presence of these specific confounding cofactors was not registered in our database; consequently, we cannot identify their role. However, these changes in LSM values, observed over time, show an opposite trajectory of fibrosis degree in around a third of SVR patients, suggesting the need for long-term monitoring of patients after SVR.

In conclusion, the present study shows the relevance of baseline LSM as a tool to stratify patients at risk of developing LRE. The dynamic LSM changes over time require, however, a continuous, long-term monitoring of this parameter to update changes in fibrosis degree also in patients obtaining SVR.

## Figures and Tables

**Table 1:** Baseline demographic and clinical characteristics of 575 cirrhotic patients who attained SVR after treatment with DAAs.

Characteristics		*n* (%)
		575
Sex	Male	305 (53.0)
	Female	270 (47.0)
Sex ratio	M/F	1.1
Age, years	≤50	83 (14.4)
	51–65	196 (34.1)
	>65	296 (51.5)
Mean age ± SD		64.1 ± 12.0
Previous IFN treatment		
	Yes	296 (51.5)
	No	279 (48.5)
Child–Pugh *		
	A	543 (94.4)
	B	32 (5.6)
LSM (kPa) **	<20	390 (71.3)
	≥20	157 (28.7)
Mean LSM ± SD ^§^		19.2 ± 7.6
Genotype	1	459 (79.8)
	other	116 (20.2)
DAAs Therapy	SOF	45 (7.8)
	SOF + other	373 (72.7)
	3D	132 (23.0)
	2D	9 (1.6)
	other	16 (2.8)
Ribavirin	Yes	222 (38.6)
	No	353 (61.4)

* None of the subject was Child–Pugh C; ** Stiffness was performed in 547 (95.1%) patients; § Kruskal–Wallis test; SOF + other = SOF + LDV, SOF + SIM, SOF + DCL, SOF + VEL; other = GLE + PIB, ELB + GRA.

**Table 2:** Absolute number and incidence rate per 100 person-years of main events during the follow-up after SVR12.

Events	*n*	Incidence Rate per 100 p/y (95% CI)
Overall death	51	1.9 (1.4–2.5)
Death for hepatic causes	17	0.6 (0.4–1.0)
Death for non-hepatic causes	34	1.3 (0.9–1.8)
Any liver decompensation *	43	1.6 (1.2–2.2)
HCC ^#^	41	1.6 (1.2–2.2)
Liver transplantation	3	0.1 (0.0–0.3)
Any event ^§^	98	3.9 (3.2–4.7)

* Ascites, hepatic encephalopathy, upper gastrointestinal bleeding, SPB; ^#^ 44% of them also experienced also liver decompensation (*n* = 18); ^§^ Includes all the events listed in the table. Events that occurred multiple times in the same subject were counted only once considering the first occurred.

**Table 3:** Factors associated with the occurrence of liver-related events (decompensation and HCC). Crude and adjusted hazard ratios (HR). Univariate and multivariate Cox model.

		Liver Decompensation				HCC			
Characteristics		HR_crude_ (95% IC)	*p*	HR_adj_ (95% IC)	*p*	HR_crude_ (95% IC)	*p*	HR_adj_ (95% IC)	*p*
Sex	F	1				1			
	M	1.0 (0.5–1.8)	0.969			2.5 (1.3–5.1)	0.008	3.6 (1.6–8.0)	0.002
Age (y)	<65	1		1		1			
	≥65	2.8 (1.4–5.7)	0.005	2.2 (1.0–4.9)	0.053	2.2 (1.1–4.2)	0.026	2.0 (1.0–4.2)	0.060
Child-Pugh	A	1		1		1			
	B	4.7 (2.1–10.5)	<0.001	1.3 (0.4–4.4)	0.635	2.6 (0.9–7.3)	0.075	2.1 (0.7–6.1)	0.169
LSM (kPa)	<20	1		1		1			
	≥20	14.8 (5.7–38.4)	<0.001	13.5 (5.2–35.3)	<0.001	10.7 (4.6–24.5)	<0.001	9.2 (4.0–21.3)	<0.001
Genotype	1	1				1			
	other	0.8 (0.4–1.8)	0.661			0.9 (0.4–2.1)	0.831		

## Data Availability

Study materials will be made available by request at University of Naples Federico II.
